# Cardiac and aerobic response to growth hormone therapy in children with short stature: a prospective study using VO_2_max and speckle tracking echocardiography

**DOI:** 10.3389/fendo.2026.1791163

**Published:** 2026-05-28

**Authors:** Ignacio Ruiz del Olmo-Izuzquiza, Antonio de Arriba-Muñoz, Lorenzo Jiménez-Montañés, Ángel Matute-Llorente, Marta Vara-Callau, Marta Ferrer-Lozano, José-Antonio Casajús-Mallén, José-Ignacio Labarta-Aizpún

**Affiliations:** 1 Pediatric Endocrinology, Hospital Infantil Universitario Miguel Servet, Zaragoza, Spain; 2 Aragon Health Research Institute (IIS Aragón), Zaragoza, Spain; 3 Pediatric Cardiology, Hospital Infantil Universitario Miguel Servet, Zaragoza, Spain; 4 Growth, Exercise, Nutrition and Development (GENUD) Research Group, Universidad de Zaragoza, Zaragoza, Spain

**Keywords:** cardiac function, growth hormone therapy, maximal oxygen uptake (VO2max), myocardial strain, short stature, speckle tracking echocardiography

## Abstract

**Introduction:**

Recombinant human growth hormone (r-hGH) enhances somatic growth and body composition, yet its short-term effects on aerobic capacity and myocardial function remain unclear in short-statured children.

**Objective:**

To assess changes in aerobic performance and cardiac morphology/function in prepubertal children with short stature undergoing r-hGH therapy, using conventional and speckle tracking echocardiography (STE).

**Material and methods:**

This prospective longitudinal study included 30 prepubertal children (18 with GH deficiency [GHD], 12 non-GHD including 6 small for gestational age [SGA] and 6 idiopathic short stature [ISS]). Participants received r-hGH for 12 months. Assessments at baseline, 6, and 12 months included anthropometry, VO_2_max (Bruce protocol), and cardiac function (including global longitudinal strain [GLS] and strain rate [GLSR] via STE). Adherence was monitored electronically.

**Results:**

r-hGH significantly increased IGF-1 and left ventricular mass indices. VO_2_max showed no overall improvement; a significant decrease was observed in GHD children, particularly girls, despite high adherence. Only GHD males exhibited supranormal VO_2_max values at baseline, and this pattern persisted at 12 months. GLSR improved significantly at 6 months in GHD patients, indicating early myocardial response to GH therapy, although GLS remained unchanged. No correlation was found between VO_2_max evolution and cardiac parameters.

**Conclusions:**

While r-hGH therapy led to favorable cardiac remodeling and early subclinical improvements in myocardial contractility in GHD children, no enhancement in aerobic capacity was observed over one year. STE proved valuable for detecting early cardiac adaptations to GH therapy. Further studies with longer follow-up and larger cohorts are warranted to clarify the functional implications of these structural changes.

## 1 Introduction

Growth is a complex, dynamic biological process regulated by the interaction between genes and environmental factors (1), directed primarily by the somatotropic axis (2). Growth hormone (GH) plays a key role in body composition (BC), muscle fiber development, and myocardial growth (3–8). Intrauterine growth restriction (IUGR) significantly influences the development of muscle tissue, potentially compromising physical performance and exercise tolerance in adulthood (9–11).

Physical exercise depends on cardiac and skeletal muscle function. GH deficiency (GHD) in adults is associated with reduced aerobic capacity (12, 13), muscular strength, and altered BC (7). Similarly, children born small for gestational age (SGA) show reduced training response and exercise tolerance (9–11). Recombinant human GH (r-hGH) therapy has been shown to improve BC and aerobic capacity (VO_2max_) in children with idiopathic short stature (ISS) (14, 15), GHD and SGA (12, 13, 16).

Regarding cardiovascular impact, r-hGH induces myocardial mass gain and improves arterial and cardiac function, likely through concentric myocardial remodelling and enhanced vascular compliance (17–19). However, early myocardial alterations may be missed by conventional echocardiographic (20), while myocardial strain assessed by 2D-speckle tracking echocardiography (STE) enables earlier detection of subclinical dysfunction (21).

This study aimed to evaluate aerobic capacity and cardiac function in short-stature children and their relationship with GH secretion and r-hGH therapy.

## 2 Materials and methods

### 2.1 Study design

A one-year prospective longitudinal study included 32 prepubertal children with short stature. All participants underwent clinical, anthropometric, biochemical, echocardiographic, and cardiopulmonary assessments at baseline, six, and twelve months of treatment.

### 2.2 Subjects

Patients, recruited from regional Pediatric Endocrinology Units, were eligible for r-hGH (Saizen**^®^**) following international guidelines, approved by the Advisory Committee for Growth Hormone and Related Products of Aragón (Spain). Exclusion criteria included age **<**6 years, chronic disease, SHOX gene mutations, interfering medications, Turner or Prader-Willi syndromes, or intellectual/motor disabilities.

Patients were classified as GHD (GH peak < 10 ng/ml in two stimulation tests) or non-GHD (NGHD), including SGA (22) and ISS (GH peak > 10 ng/ml). Two patients were excluded: one due to incomplete follow-up and another due to improper completed consent documentation.

### 2.3 Anthropometric measures

Height (centimetres) was measured in standing position using a Harpenden stadiometer (Holtain Ltd., UK), and weight (kilograms) with an AGI scale (IMSA, Spain). The body mass index (BMI) was calculated as weight(kg)/height(m)^2^. Growth velocity (GV) was recorded in cm/years. Birth weight (BW) (kg) and birth length (BL) (cm) were obtained from medical records. All parameters were compared to the Spanish reference population using standard deviation scores (23).

### 2.4 Aerobic capacity

Maximal oxygen uptake (VO_2max_, mL/kg/min) was determined via breath-by-breath gas analysis using an open-circuit spirometry (Oxycon Pro, Jaeger/Viasys Healthcare, Germany), with values averaged every 15 seconds. Daily calibration was performed with a known gas and volume as recommended by the manufacturer. Heart rate was continuously monitored with a 12-lead ECG system (H12+, Mortara Instrument, USA). Patients underwent treadmill exercise (Quasar Med 4.0; h/p/cosmos, Germany) following the standardised Bruce protocol (12), under medical supervision by a sports medicine physician, who also ensured safety and fitness to maximal effort. Results were compared with the reference values of Chillón et al. (24), based on a cohort of more than 2,000 school-aged children and adolescents from both urban and rural areas of the region.

### 2.5 Echocardiography

Cardiac evaluations were performed in supine decubitus and left lateral decubitus positions using a Siemens Acuson SC2000 ultrasound system (Siemens Healthcare, Germany) equipped with an 6 and 3 Mhz straight-line transducer. The assessment included: left ventricular mass (LVM), gr; LVM index (LVMI), gr/m^2^; left ventricle tele-diastolic volume (LVTDV), mL; LVTDV index (LVTDVI), ml/m^2^. Speckle-tracking determinations were carried out by the same Pediatric Cardiologist. Offline strain analysis on apical loops with Velocity Vector Imaging (VVI) 3.0 (Siemens) software was performed. The cardiologist manually traced the endocardium in end-diastole. The movement of the myocardial wall (from endocardium to epicardium) was manually drawn and therefore defined the areas of interest. LV wall was split automatically into 6 segments by the VVI software, using these segments for measuring the LV strain. The following LV STE parameters were recorded: global longitudinal strain (GLS) in % (percentage of longitudinal shortening of the left ventricle during systole (25)) and global longitudinal strain rate (GLSR) in seg^-1^ (rate at which this deformation occurs (25)). Strain data were shown in absolute values due to the lack of standardized pediatric reference of Z-score values at the time of the study.

### 2.6 Adherence

Adherence was calculated as the percentage of days the prescribed dose was administered (26) using the easypod™ device, which records both administered and missed injections. Adherence at month 12 was categorized based on Cutfield et al. (27): good (≥92%), moderate (86-91%) and poor (≤85%). For statistical analysis, patients were classified as adherent (≥92%) or non-adherent.

### 2.7 Statistical analysis

Statistical analysis was performed using SPSS version 22 (IBM Corp., USA). Quantitative variables were presented as mean ± SD, qualitative variables as absolute and relative frequencies. The GHD group was compared with the combined non-GHD (NGHD) group, and subsequently comparisons within the NGHD subgroups (SGA and ISS) were performed. Additionally, longitudinal comparisons were conducted between baseline and 12-month values within each group using paired statistical tests (paired Student’s t-test or Wilcoxon signed-rank test), as appropriate depending on data distribution. Student’s t-test and Mann-Whitney U test were used for comparisons between independent groups. Pearson or Spearman correlation assessed associations of quantitative variables, depending on distribution. Categorical comparisons were analyzed with Fisher’s exact test. A General Linear Model (GLM) for repeated measures was used to analyze parameters collected at three different points in time (baseline, month 6, and month 12), only patients with complete data across all visits were included in this model. Statistical significance was set at 5%.

## 3 Results

### 3.1 Clinical characteristics

Eighteen GHD (12 female, 8.5 ± 1.8 years) and 12 NGHD (10 female, 8.6 ± 1.9 years) children were included, all prepubertal (Tanner I). The NGHD group included six SGA and six ISS patients. Four participants (13.3%) were non-adherent to treatment. **Table 1** summarizes the general characteristics of the cohort, and **Table 2** details subgroup differences.

**Table 1:** Clinical and hormonal parameters, growth hormone dose, and maximal oxygen uptake over the follow-up period in the study cohort.

Study parameter	Total (n=30)		
	Baseline	6 months	12 months
BW (SD)	-0.72(1.07)		
BL (SD)	-0.9(1.24)		
Height (SD)	-2.8 (0.6)	-2.4 (0.6)	-2.2 (0.6)
GV (SD)	-1.6 (2.9)	3.1 (2.7)	2.8 (2.6)
Weight (SD)	-1.8 (0.6)	-1.7 (0.5)	-1.6 (0.6)
Z score BMI (SD)	-0.685 (0.880)	-0.813 (0.885)	-0.739 (0.960)
r-hGH Dose (mg/kg/day)	0.031	0.031	0.028
IGF-1 (ng/ml)	131.02 (55.24)	206.43 (60.96)	286.79(114.81)
VO_2max_ (mL/kg/min)	49.3 (4.9)	48.9 (5.1)	47.1 (5.0)

BL, birth length; BMI, Body Mass Index; BW, birth weight; GV, growth velocity; IGF-1, insulin like growth factor 1; r-hGH, recombinant human Growth Hormone; SD, Standard Deviation; VO_2max_, Maximal Oxygen Uptake.

**Table 2:** Clinical and hormonal parameters, growth hormone dose, and maximal oxygen uptake over the follow-up period in the study subgroups.

Study parameter	GHD (n = 18)			NGHD (n = 12)					
				SGA (n = 6)			ISS (n = 6)		
	Baseline	6 months	12 months	Baseline	6 months	12 months	Baseline	6 months	12 months
BW (SD)	-0.45 (0.85)			-1.96 (1.15)*			-0.30 (0.72)*		
BL (SD)	-0.69 (1.11)			-2.13 (1.40)**			-0.35 (0.63)**		
Height (SD)	-2.60 (0.50)	-2.30 (0.70)	-2.10 (0.50)	-3.22 (0.81)	-2.55 (0.49)	-2.61 (0.91)	-2.91 (0.41)	-2.21 (0.21)	-2.06 (0.51)
GV (SD)	-1.70 (2.50)	2.80 (2.20)	2.40 (2.00)	-1.30 (5.09)	3.33 (2.46)	3.17 (3.82)	-1.48 (1.66)	3.84 (4.12)	3.65 (2.81)
Weight (SD)	-1.70 (0.60)	-1.60 (0.60)	-1.50 (0.60)	-2.21 (0.35)	-2.04 (0.42)	-2.03 (0.51)	-1.81 (0.42)	-1.59 (0.44)	-1.63 (0.73)
Z score BMI	-0.56 (0.98)	-0.63 (0.90)	-0.59 (0.90)	-0.56 (0.98)	-0.63 (0.90)	-0.59 (0.90)	-0.88 (0.70)	-1.09 (0.80)	-0.96 (1.00)
GH dose (mg/kg/day)	0.029	0.03	0.025	0.035	0.035	0.035	0.029	0.032	0.031
IGF-1 (ng/ml)	135.60 (45.80)	210.20 (64.80)	305.20 (135.60)	156.86 (93.90)^§^	208.60 (73.410)	260.00 (73.41)^§^	102.98 (32.74) *^¶^*	196.33 (50.25)	252.4 (44.92) *^¶^*
VO_2max_ (mL/kg/min)	50.20 (5.10)^†^	48.90 (5.70)	47.50 (5.20) ^†^	47.57 (5.32)	46.93 (4.60)	46.45 (6.46)	48.48 (4.12)	50.75 (3.35)	47.05 (3.59)

BL, birth length; BMI, Body Mass Index; BW, birth weight; GHD, growth hormone deficiency; GV, growth velocity; IGF-1, insulin like growth factor 1; NGHD, non GHD; r-hGH, recombinant human Growth Hormone; SD, Standard Deviation; VO_2max_, Maximal Oxygen Uptake.

^*^ p = 0.0152 and ^**^p = 0.0411: comparison of BW and BL respectively between SGA and ISS at baseline.

^§^ p = 0.0011 and ^¶^p < 0.0001: comparison of IGF-1 levels between baseline and 12 months in SGA and ISS respectively.

^†^ p = 0.04: comparison of VO2max levels between baseline and 12 months in GHD group.

Among GHD children, the mean GH peak after stimulation was 6.44 ± 1.9 ng/ml, with only one participant below 3 ng/ml. BW and BL differed significantly between SGA and ISS subgroups. IGF-1 levels increased significantly from baseline to month 12 in both the SGA and ISS groups; however, no significant differences were found between the three diagnostic categories. Although SGA patients had higher IGF-1 levels than ISS at month 12, the difference was not statistically significant. No differences were observed by gender or diagnostic group in any other baseline parameter.

### 3.2 Aerobic capacity

No significant changes in VO_2max_ were observed between baseline (49.3 ± 4.9 mL/kg/min) and 12 months (47.1 ± 5.05) across the study population (p = 0.371). At baseline, VO_2max_ was 52.8 ± 5.0 mL/kg/min in boys and 48.1 ± 4.4 in girls. After one year, values remained stable in boys (52.8 ± 3.1) but decreased in girls (45.2 ± 4.0), although sex differences were not statistically significant within the overall group. Compared to the reference population (24), both males (p = 0.003) and females (p = 0.02) had higher baseline VO_2_max values. At month 12, this trend persisted only in boys (p = 0.003).

Subgroup analysis at baseline showed that in GHD children, males had a VO_2max_ of 55.0 ± 3.2 mL/kg/min, while females reached 47.8 ± 4.1. In the NGHD group, males and females exhibited values of 46.2 ± 2.2 and 48.4 ± 4.9, respectively. Among these groups, only GHD males demonstrated significantly higher values compared to the reference population (24)(p < 0.001).

**Figure 1:**
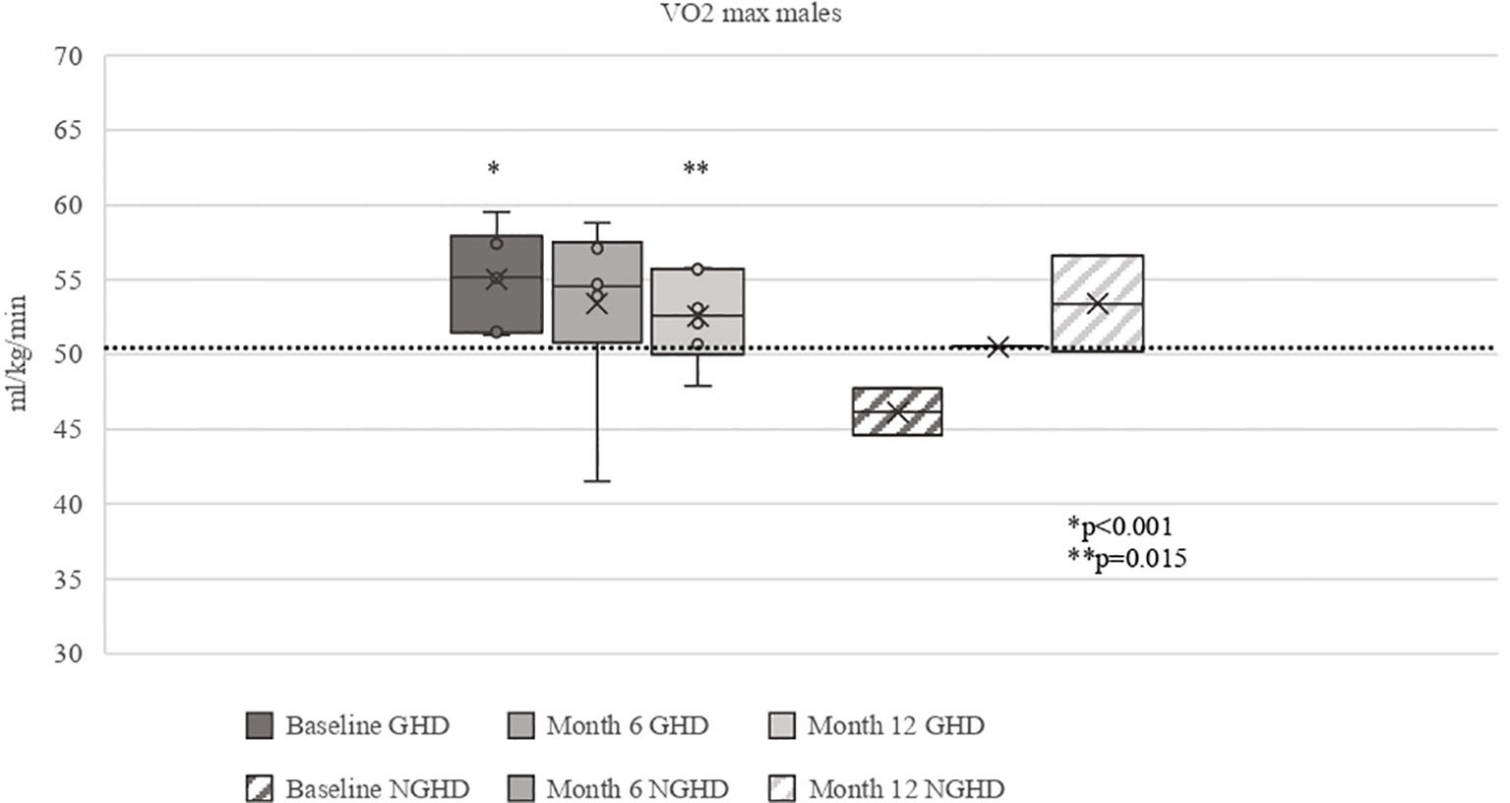
Evolution of aerobic capacity (VO_2_max) over the study period in male patients with growth hormone deficiency (GHD) and non-GHD, compared to the reference population (27) (dotted line). *p<0.001, **p=0.015.

No significant differences in VO_2max_ progression were found between diagnostic groups (p = 0.585), or between SGA and ISS subgroups (p = 0.631). Within the GHD children, aerobic capacity decreased significantly after one year of treatment compared to baseline (p = 0.046). GHD males exhibited significantly higher VO_2_max values compared to the reference population (24) both at study entry (55 ± 3.2 mL/kg/min) and month 12 (52.5 ± 3.0 mL/kg/min), as illustrated in **Figure 1**. No differences were found among girls nor NGHD patients.

Given the generally high adherence rate, additional exploratory analyses were conducted using different adherence thresholds (95%, 96%, 97%, 98%, and 99%) to assess potential associations with VO_2max_ changes. No significant associations were detected at any cut-off level.

### 3.3 Echocardiography

Echocardiographic parameters are summarized in **Table 3**. The analysis, based on the 21 patients with compete data at all time points, revealed a significant increase in LVM (p < 0.05), LVMI (p = 0.031) and LVTDV (p = 0.03), from baseline to month 12 in the entire cohort.

**Table 3:** Echocardiographic parameters of cardiovascular function in the study population, stratified by diagnostic group (n=21).

Cardiac parameter	GHD (n = 13)			NGHD (n = 8)					
				SGA (n = 4)			ISS (n=4)		
	Baseline	6 months	12 months	Baseline	6 months	12 months	Baseline	6 months	12 months
LVM (gr)	33.58 (13.59)	37.96 (10.89)	44.41 (12.97)	29.95 (8.95)	32.73 (11.66)	39.95 (11.35)	42.35 (10.28)	35.56 (11.11)	49.25 (13.22)
LVM INDEX (gr/m^2^)	39.67 (11.37)	43.16 (8.35)	46.97 (6.55)	40.29 (16.76)	40.91 (13.42)	47.98 (12.83)	44.80 (6.75)	35.49 (7.68)	46.45 (9.57)
LVTDV (mL)	49.41 (14.49)	46.55 (14.02)	50.78 (13.00)	35.00 (12.52)	40.23 (8.03)	46.23 (11.82)	41.48 (13.09)	47.13 (10.05)	56.34 (5.78)
LVTDVI (mL/m^2^)	59.16 (14.13)	52.87 (13.15)	54.07 (6.33)	47.25 (21.61)	51.14 (12.88)	56.08 (16.45)	43.79 (11.740)	47.37 (5.44)	55.34 (5.78)
GLS (%)	-21.86 (2.66)	-21.53 (2.48)	-21.25 (2.78)	-20.73 (2.06)	-22.47 (1.79)	-23.24 (2.09)	-22.77 (0.98)	-22.38 (1.57)	-20.78 (2.33)
GLSR(seg-1)	-1.86 (0.15)	-1.82 (0.24)	-1.73 (0.24)	-1.70 (0.49)	-1.84 (0.12)	-1.97 (0.25)	-1.85 (0.17)	-1.94 (0.16)	-1.60 (0.08)

GLS, global longitudinal strain; GLSR, global longitudinal strain rate; LVM, left ventricular mass; LVMI, left ventricular mass index; LVTDV, left ventricle tele-diastolic volume; LVTDVI, left ventricle tele-diastolic volume index.

**Figure 2:**
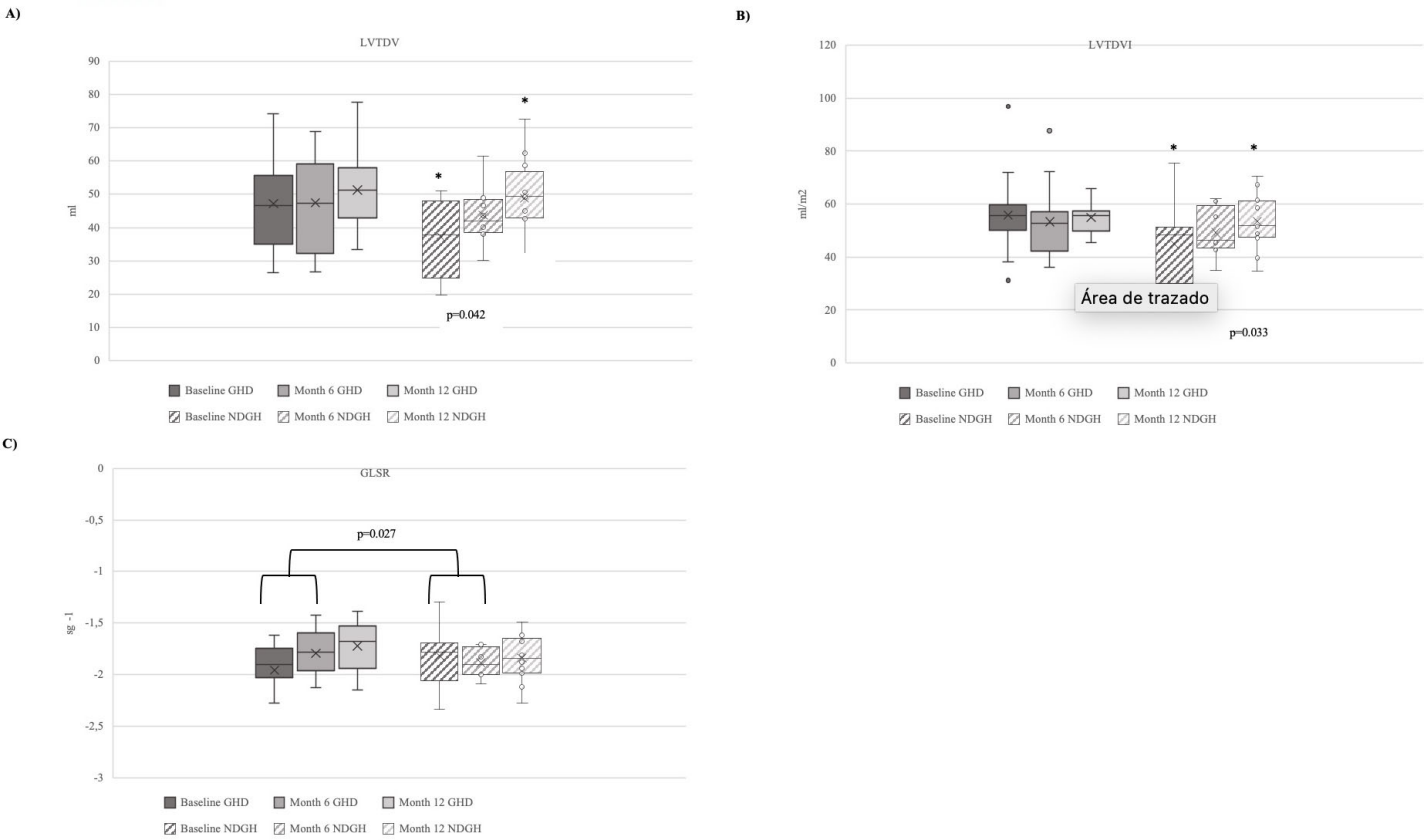
Cardiovascular function according to diagnostic groups: left ventricle telediastolic volume **(A)**, left ventricle telediastolic volume index **(B)** and global longitudinal strain rate **(C)**. * corresponds to p values in both **(A, B)**.

NGHD patients showed a more marked increase in LVTDV, both in absolute and indexed values (**Figures 2A, B**). When analyzed by subgroups, both ISS (p = 0.026) and SGA (p = 0.0353) children exhibited significant increases in LVTDV over time. However, no differences were found between the two subgroups in absolute or indexed volume.

During the first six months of treatment, GHD children showed a significantly greater increase in GLSR compared to NGHD (**Figure 2C**). These differences were not sustained at month 12. No significant changes were observed in GLS values at any time point in either group. Similarly, when comparing GLSR values between SGA and ISS children, no significant differences were identified. Moreover, no correlations were found between changes in VO_2max_ and other cardiac function parameters in either group throughout the study period.

## 4 Discussion

This study provides an in-depth evaluation of the impact of r-hGH therapy on aerobic capacity and cardiac function in children with short stature, including both GHD and NGHD groups. The findings not only corroborate existing evidence but also bring new insights into the complex physiological effects of r-hGH in this population.

The administration of r-hGH led to an increase in IGF-1 levels in both the GHD and NGHD groups, with a more pronounced response observed in the GHD cohort. The partial deficiency in the vast majority of the GHD patients could explain the lack of significant differences in IGF-1 levels between groups.

Unlike previous studies, our cohort did not show an increase in VO_2max_. Furthermore, there was a significant decrease in GHD individuals at month twelve. NGHD males had higher VO_2max_ values than the reference population, both at baseline and month 12. No changes were found in NGHD children. These results can be attributed, first, to the heterogeneity of our sample and, second, to the fact that GHD patients did not exhibit a severe deficiency (normal mean values of baseline IGF-1). These facts may explain their comparable VO_2max_ values to those of the reference population.

The determination of VO_2max_ is widely recognized as the gold standard for assessing aerobic functional capacity. GH has been proposed to enhance aerobic performance through multiple mechanisms: increased in oxygen-binding capacity in blood, improved myocardial output, changes in substrate metabolism, and increases in lean body mass (LBM) (7). These physiological effects underscore the importance of GH in modulating aerobic performance, particularly in populations with GH deficiencies or growth impairments. Evidence supporting the efficacy of r-hGH treatment in improving VO_2max_ is robust. Two case-control studies in children with GHD (28, 29) and two meta-analysis in adults (30, 31) have consistently reported lower baseline VO_2max_ values in GHD patients, followed by significant improvements after 12 months of r-hGH therapy. Furthermore, several clinical trials have explored the relationship between r-hGH treatment and VO_2max_. In adult patients following cranioencephalic trauma, VO_2max_ values were significantly lower in those with total or partial GHD compared to individuals with normal GH levels (12). Comparable reductions have been reported when comparing VO_2max_ in GHD patients with healthy subjects (12, 13). These findings highlight the impact of GHD on aerobic capacity.

In addition to GHD, IUGR plays a fundamental role in determining the quantity and quality of muscle tissue during infancy, with long-term consequences for exercise capacity and fatigue tolerance in adulthood. Regarding VO_2max_ in SGA subjects, the available literature remains limited. Crispi et al. reported a 12% reduction in oxygen consumption at peak exercise among young adults born SGA compared to those born appropriate for gestational age (AGA) (32). Similarly, a case-control study involving 22 SGA children found comparable reductions in aerobic capacity (33). Balasekaran et al. specifically evaluated the relationship between r-hGH treatment and VO_2max_ in short children, concluding that growth induced by GH therapy results in a statistically significant increase in VO_2max_, attributed to the associated increase in fat-free mass (16). Our results showed that the VO_2max_ progression did not differ significantly between GHD and NGHD groups, or between the SGA and ISS subgroups, suggesting uniform effects of r-hGH across these cohorts.

As previously discussed, our findings did not align with the expected outcomes. Several factors may explain this. First, the study design was based on comparisons with a reference population rather than a case-control approach. Second, participants did not present severe growth hormone deficiencies. Finally, most cases included idiopathic short stature or partial GH deficiencies, which may account for the minimal differences observed with the reference population.

Early infancy represents a crucial period for LVM development, during which cardiomyocytes respond to changes in pressure or volume load, as well as to hormonal factor such as GH and IGF-1 (34). Studies have demonstrated that LBM and BW are positively correlated with LVM during childhood (35). Consequently, conditions that impair growth, such as GHD and IUGR, can have long-term repercussions on cardiac morphology and function.

The effects of r-hGH therapy on cardiac performance and morphology have been well documented in GHD patients. Multiple studies report a significant increase in LVM when indexed to body surface area after initiation of GH therapy. Capalbo et al. observed a notable improvement in LVM alongside enhanced myocardial contractility after 12 months of treatment (20, 28). These favorable changes are believed to reflect cardiac remodeling mediated by GH-induced IGF-1 stimulation, which promotes hypertrophy of myocardial tissue without inducing pathological remodeling (18). While z-scores are valuable for the assessment of individual patients, we considered that cohort-level changes before and after treatment are more appropriately captured using absolute values and, particularly, parameters normalized to body weight and body surface area. However, this approach may not fully distinguish between physiological growth and treatment-related cardiac remodeling.

In line with these findings, our study also demonstrated a significant increase in LV parameters after r-hGH therapy, particularly in GHD children. Importantly, GLSR, a sensitive marker of myocardial contractility evaluated through STE, showed a significant improvement during the first six months of therapy in GHD patients compared to NGHD. This suggests an early adaptive response of the myocardium to GH stimulation, possibly linked to enhanced myocardial growth and contractility. While conventional echocardiography is limited in detecting subclinical myocardial dysfunction, STE provides a more nuanced evaluation of cardiac deformation mechanics. Our findings underscore the importance of incorporating advanced imaging techniques, such as STE, to capture early myocardial changes that may not yet translate into measurable differences in global systolic or diastolic function. Our results indicate that GLSR is an early marker of myocardial improvement in GHD children receiving r-hGH therapy. These findings are consistent with prior studies showing that GH therapy improves circumferential fiber shortening and reduces end-systolic stress, both of which are load-independent markers of contractility (20). By incorporating STE into the assessment of cardiac function, we were able to identify early myocardial adaptations that may precede detectable changes in global systolic performance. To our knowledge, this study is the first to demonstrate the utility of STE in comparing cardiac responses to GH therapy between GHD and NGHD children, highlighting its role as a sensitive tool for evaluating subclinical cardiac dysfunction. While GLSR improved significantly in GHD patients, no comparable changes were observed in NGHD children, suggesting that the impact of GH therapy on myocardial contractility may be more pronounced in children with a hormonal deficiency rather than those with constitutional growth impairments.

Our study found no significant correlation between VO_2max_ improvements and cardiac structural changes, despite increased LVM in GHD patients. While Capalbo et al. suggested that ventricular enlargement enhances aerobic capacity (20), other studies, including ours, did not confirm this link (18). Instead, VO_2max_ improvements may result from increased LBM, peripheral oxygen utilization, and muscle efficiency. r-hGH therapy enhances oxygen consumption and workload capacity primarily through LBM gains and fat mass reduction (18). The absence of VO_2max_ improvements in our study may reflect a short follow-up or gender-specific differences. The observed decrease in VO2max in girls should be interpreted cautiously. This finding may reflect the small size sample and sex imbalance, as well as supranormal VO_2max_ values found in males with GHD. To the best of our knowledge, the existing literature does not specifically address a sex-related decline in VO_2max_ during GH therapy in pediatric population.

Data on cardiac performance in SGA children remain limited. Adults born SGA have demonstrated subtle cardiac alterations, such as increased left ventricular end-diastolic and end-systolic diameters, which were not significant when indexed to body surface area or height (35, 36). Aurensanz et al. reported similar findings, suggesting that IUGR leads to latent subclinical changes in cardiac contractility, which may persist into adulthood (37). Our results showed that NGHD patients, including SGA and ISS subgroups, exhibited minimal changes in LVM and strain parameters following GH therapy, in contrast to the clear improvements seen in deficient patients. These finding highlight a potential difference in myocardial responsiveness to GH therapy between NGHD and GHD population, which could be attributed to underlying differences in muscle tissue development and cardiomyocyte number resulting from IUGR.

The strengths of our study include its prospective design, comprehensive evaluation of aerobic and cardiac parameters, and well-defined subgroup stratification. Advanced echocardiographic techniques and robust statistical methods add rigor to the findings. A structured adherence measurement system enhances reliability. However, the small sample size limits statistical power and generalizability. The short follow-up may have prevented detecting long-term effects of r-hGH therapy. Future research should include advanced imaging, larger cohorts, and extended follow-up. Baseline heterogeneity, such as sex distribution and initial cardiac or aerobic status, may have influenced results. This underscores the need for individualized GH therapy evaluation in pediatric patients.

In conclusion, our study highlights that while r-hGH therapy induces significant improvements in growth and cardiac morphology, its effects on aerobic capacity remain limited in the short term. Our study emphasizes the importance of STE as a tool for detecting early myocardial changes and provides valuable insights into the differential effects of GH therapy on cardiac function in GHD and NGHD populations. Further investigations incorporating larger cohorts and advanced imaging modalities will help refine our understanding of GH therapy’s impact on cardiac and cardiopulmonary function in children with growth impairments.

## Data Availability

The datasets presented in this article are not readily available because the datasets are not publicly available due to ethical restrictions but are available from the corresponding author upon reasonable request and approval. Requests to access the datasets should be directed to jiruizdelolmo@salud.aragon.es.
